# Unsupervised Conditional Diffusion Models in Video Anomaly Detection for Monitoring Dust Pollution

**DOI:** 10.3390/s24051464

**Published:** 2024-02-23

**Authors:** Limin Cai, Mofei Li, Dianpeng Wang

**Affiliations:** 1 School of Mathematics and Statistics, Beijing Institute of Technology, Beijing 100081, China; 2 Inner Mongolia Ecological Environment Big Data Co., Ltd., Hohhot 010070, China; limofei@crcept.com

**Keywords:** conditional diffusion models, dust pollution, latent compression, video anomaly detection

## Abstract

Video surveillance is widely used in monitoring environmental pollution, particularly harmful dust. Currently, manual video monitoring remains the predominant method for analyzing potential pollution, which is inefficient and prone to errors. In this paper, we introduce a new unsupervised method based on latent diffusion models. Specifically, we propose a spatio-temporal network structure, which better integrates the spatial and temporal features of videos. Our conditional guidance mechanism samples frames of input videos to guide high-quality generation and obtains frame-level anomaly scores, comparing generated videos with original ones. We also propose an efficient compression strategy to reduce computational costs, allowing the model to perform in a latent space. The superiority of our method was demonstrated by numerical experiments in three public benchmarks and practical application analysis in coal mining over previous SOTA methods with better AUC, of at most over 3%. Our method accurately detects abnormal patterns in multiple challenging environmental monitoring scenarios, illustrating the potential application possibilities in the environmental protection domain and beyond.

## 1. Introduction

Coal generates more than a third of the world’s electricity, which is a major source of air pollution and causes damage to public health during its mining and combustion processes. One study showed that suspending coal mining in countries such as China, which has long been the global leader in coal production, may reduce local air pollution by 8%. Since we still rely on coal, it is necessary to strictly monitor and control the generation of particulate matter during coal production and storage to reduce the environmental impact of coal production. In China, to support environmental protection and law enforcement, video surveillance systems are installed in coal mining, transportation, storage and washing. The videos collected from these surveillance systems are used to monitor abnormal harmful dust pollution and issue timely warnings, which can effectively improve the law enforcement efficiency of relevant departments.

Generally, monitoring abnormal dust pollution is equivalent to video anomaly detection (VAD), which is popular but complex. Two kinds of approaches are prominent in the literature in this field: weakly supervised and unsupervised methods [1]. Weakly supervised methods model the abnormal probability of instances relying on labels [2,3,4]. Sultani et al. [5] divided the video into several isometric temporal segments and detected anomaly segments with high anomaly scores through a C3D network. Tian et al. [6] proposed a network based on dilated convolutions and self-attention mechanisms to capture the temporal dependencies of long and short ranges. However, existing weakly supervised methods in VAD face some constraints [7]. Since the frequency of abnormal events is very low, the training data is always extremely unbalanced, which poses a significant challenge for model building. Besides, abnormal events that do not frequently appear in the training stage may occur in the monitoring stage, raising the challenge of recognizing new abnormal events.

To cope with these challenges, unsupervised methods have been proposed in recent years. Until now, there are two kinds of mainstream unsupervised methods [8]. One kind tries to build the prediction model based on the relationship between frames and detect abnormal events based on the differences between the predictions and true observations; larger differences mean a higher anomaly likelihood. In this way, Long Short-Term Memory (LSTM) and Recurrent Neural Network (RNN) were always utilized to extract important information about the context from the existing frames [9,10,11]. Inspired by Natural Language Processing (NLP), a vision transformer (ViT) was proposed which divided the videos into patches of the same size and encoded these patches into embedding sequences to learn about the correlation between features [12,13,14].

The other kind is based on the generative strategy. For these methods, a generative model is trained to mimic the distribution of normal videos, and the observed videos are reconstructed based on the generative model. Kingma et al. [15] employed a convolutional neural network named Variational Autoencoder (VAE) to simulate the distribution of the latent variables, by which the images are generated. Hasan et al. [16] extracted appearance and motion flows manually and input them into VAE, which makes the model learn about the inner features of data better. Liu et al. [17] took temporal features into consideration and proposed a a spatial-temporal autoencoder to learn about the spatial and temporal patterns of the data. The most popular method is called the Generative Adversarial Network (GAN), which includes a generator to create videos and a discriminator to classify the authenticity of videos. Based on GANs, Zaheer et al. [18] assembled an autoencoder generator network to generate the instance and a fully connected discriminator network to classify the instance with a binary pseudo label. Huang et al. [19] proposed a temporal contrastive network to capture the semantic features and tackle anomaly detection. Li [20] used the video context to generate continuous frames before and after a given frame, and compared them with ground truths.

Even though the existing generative methods perform greatly in unsupervised learning and have become mainstream in VAD, they still cannot reach the desired performance since there are no labels to directly classify anomalies—only the video data is available for learning. The models must learn about the underlying features of massive data and optimize the approximation of the intrinsic distribution to generate realistic instances, which poses higher requirements on the model structures. Additionally, generating instances based on features and probability distributions cannot guarantee the robustness of the model; it may cause misidentifications in different datasets, which is fatal in VAD. It is also difficult to define abnormal events with a clear and uniform criterion, which brings about the challenge of judging whether the video is abnormal. To date, there is no solution to this problem.

Currently, a new kind of probabilistic-based generative model called the diffusion model has become more and more popular in the field of computer vision. Hoppe et al. [21] proposed the Denoising Diffusion Probabilistic Model (DDPM) based on diffusion models to generate high-quality images. After continuous explorations and refinements of DDPM [22,23,24], Dhariwal et al. [25] found it outperformed mainstream state-of-the-art (SOTA) GANs in image synthesis, which is the most outstanding achievement of diffusion models. In addition to generation, the diffusion models have been applied to anomaly detection. In the medical domain, diffusion models are used to detect physical illnesses such as brain tumors. Wolleb et al. [26] reconstructed MRIs through diffusion models and recognized abnormal MRIs with high errors between reconstructions and inputs. Finn et al. [27] divided a brain MRI scan into several patches and reconstructed them separately to improve the accuracy of the model.

Diffusion models still face some constraints in their application in VAD, despite their significant strengths. First, although attempts have been made to apply the model to the video-generation domain [28,29,30,31], it is more commonly applied to image-related fields and is rarely considered in in more complex fields like videos, especially in VAD. Compared with images, video sequences require additional consideration of the relationships between frames. Therefore, exploring how to fully utilize the extra temporal features in videos and generate sequences to a high quality is worthy of investigation. Second, current methods are more suitable for anomaly detection in single scenarios. When faced with different types of normal videos in complex scenarios, which are common in practical VAD applications, they may fail to accurately generate the required instances. Furthermore, due to the mechanism of diffusion, high computation costs make it difficult to widely apply the models to various practical fields, especially online monitoring, and for the models to be accepted by most enterprises due to budget constraints.

In this study, we introduce a new unsupervised method based on latent conditional diffusion in VAD. We introduce the overall framework for video types and propose several improvements to the structure. We also apply our method to different monitoring datasets. Using data collected from videos, our algorithm detects anomalies in various scenarios effectively. The main contributions of our study are summarized as follows:

1. A new unsupervised diffusion-based method is proposed by considering the monitoring of the environment. Experimental results on popular benchmarks illustrate the superior performance of the proposed method. A case study in coal mining based on real data from surveillance systems aiming to detect harmful dust pollution shows that our algorithm can be used to develop cleaner and more sustainable practices to reduce the environmental impact of the industries;
2. We propose a spatio-temporal structure in the network architecture. The structure considers temporal features in videos and combines them with spatial features through different attention blocks. Additionally, we propose a new conditional mechanism and apply it to the network with convolutional layers. The mechanism divides the input video into equal-length bins and uniformly samples frames from each bin to guide sequence generation. These designs help for learning about the distributions of multiple normal scenarios well and generate high-quality reconstructions close to the original sequences;
3. A novel latent compression strategy is proposed based on a pre-trained autoencoder to reduce computation costs by compressing the videos into a lower dimensional space. We modify the training loss by removing the Kullback–Leible divergence and replacing it with min–max normalization. This strategy successfully compresses the videos to a high quality while preserving the original inner features.

The remainder of this article is organized as follows: In Section 2, the details of our method and related improvements in the framework are proposed. Some numerical experiments based on the public benchmarks are provided in Section 3 to assess the performance of our new method. In Section 4, a real data case study about coal-mining dust detection is given. Our conclusions and future work are summarized in the last Section 5.

## 2. Methodology

In this section, our new method is presented in detail. Before that, the traditional DDPM is briefly reviewed first. Then, our suggested improvements and modifications for the method are proposed.

### 2.1. Review of the DDPM

As a classical type of diffusion model, the DDPM tries to simulate the thermodynamic processes in physics to learn about the inner features and the distribution of instances. It consists of two parts: a forward Markov diffusion process and a backward reversed process. The diffusion process progressively adds noise to data until it is corrupted and close to the isotropic Gaussian distribution. The reversed process approximates the distribution of data during each noise addition in the diffusion process and denoises it to generate new samples that highly follow the original distribution.

Let $x_{0}∼q\left(\cdot \right)$ be the original input and T∈N be the number of total forward steps. Typically, in the diffusion process, given the number of total forward steps, with adding random standard Gaussian noise ϵ∼N(0,I) by a variance schedule $\beta_{t}\in \left(0,1\right)$ step by step, noisy variables $x_{1},x_{2},\ldots ,x_{t}$ follow distributions such as

(1) $$q\left(x_{t}|x_{t-1}\right)=N\left(x_{t};\sqrt{1-\beta_{t}}x_{t-1},\beta_{t}I\right),$$

where 1≤t≤T, $\beta_{t}\in \left(0,1\right)$ denotes a variance schedule and I is the identity matrix. By shifting the distribution of $x_{0}$ to $x_{t}$ with the re-parameterization, the output after *T* steps in the diffusion process is

(2) $$x_{T}=\sqrt{\bar{\alpha_{T}}}x_{0}+\sqrt{1-\bar{\alpha_{T}}}\epsilon ,$$

where $\bar{\alpha_{T}}=\prod_{i=1}^{T}\left(1-\beta_{i}\right)$.

After forward diffusion, a Markov-reversed process starts from $x_{T}$ to generate great samples based on the previous diffusion process, which tries to approximate the distribution of $x_{t-1}$ given $x_{t}$ as

(3) $$p_{\theta }\left(x_{t-1}|x_{t}\right)=N\left(x_{t-1};\mu_{\theta }\left(x_{t},t\right),\beta_{t}I\right),$$

where $\mu_{\theta }$ is the distribution expectation to be trained with unknown parameters θ. By minimizing a variational lower bound (VLB) of the negative log-likelihood, we can obtain the simulated distribution close to the real one and take samples in each reversed step from it. The objective is formulated as

(4) $$\begin{array}{cc} L= & -\log p_{\theta }\left(x_{0}|x_{1}\right)+D\left(q\left(x_{T}|x_{0}\right)||p\left(x_{T}\right)\right)\\ \; & +\sum_{t>1}D\left(q\left(x_{t-1}|x_{t},x_{0}\right)||p_{\theta }\left(x_{t-1}|x_{t}\right)\right), \end{array}$$

where D denotes Kullback–Leible divergence between two distributions and $p_{\theta }$ denotes the simulated distribution in the reversed process. After *t* times of sampling from the simulated distribution in each reversed step, the final generated sample which is highly similar to original data can be obtained.

It is obvious that videos have an additional time dimension compared to images. For convenience, allow a video sequence $x_{0}=\left[x_{0}^{1},x_{0}^{2},\ldots ,x_{0}^{N}\right]\in R^{N\times C\times H\times W}$, where $x_{0}^{i}$ denotes the *i*th frame in the video and N,C,H,W denote the number, channels, heights and widths of frames, respectively.

### 2.2. Spatio-Temporal Structure through Diffusion

Images are basic units of visual scenes in computer vision. Most methods, including DDPMs, are based on images. Different from a single image, video sequences are composed of multiple consecutive frames. It is certain that there are strong correlations among these frames. Therefore, temporal correlations are seen as equally important as spatial correlations in videos. However, current diffusion-based methods rarely consider the importance of the temporal aspect, which treats frames in a video sequence as a batch of images and builds the network with spatial-only convolutions. Some methods simply improved the structures using 3D convolutions [30], but they required more parameters and were much more difficult to train. Some attempted to sequentially extract features of data from spatial and temporal dimensions with full connections [32], which still increased the complexity of processing and resulted in the loss of key features from different dimensions to a certain extent. These changes do not significantly improve the performances of the models.

In this study, we propose a spatio-temporal factorized U-Net architecture to capture both spatial and temporal features in video sequences. The architecture consists of two paths, with an n-layer down-sampling path capturing contextual information and the same n-layer up-sampling path reconstructing the resolution.

In each layer, we propose a spatio-temporal structure composed of multi-head self-attention sub-blocks with adjustable parameters, which focus on feature extractions on both spatial and temporal dimensions. To prevent features of different dimensions from interfering with each other, for the input $x_{t}$, we have it pass through two embedding attention networks Spa(·) and Temp(·) for spatial and temporal analysis, respectively. To comprehensively consider two kinds of features and measure the weight between them adaptively, we use two learnable coefficients a and b and add the outputs through two embedding networks as

(5) $$x_{t}=aSpa\left(x_{t}\right)+bTemp\left(x_{t}\right).$$

This way of separation greatly preserves the input feature information in each dimension. With the addition of outputs through spatial and temporal attention, the network merges and learns about the comprehensive inner features, which consider both spatial and temporal correlations in a continuous sequence.

### 2.3. Conditional Self-Guidance on Videos

The proposed spatio-temporal structure makes better use of temporal information and strengthens the connections between spatial and temporal features in videos. However, it still cannot reach the goal of the accurate recognition of abnormal videos. In unsupervised VAD, we aim to compare differences between inputs and reconstructions, which have more requirements on outputs through the model. One of the exposed constraints of the DDPM is that the model cannot control the specific content or class we want to generate. While faced with various scenarios, the model may also generate various normal sequences which totally differ from inputs, and cause incorrect anomaly alarms.

In this study, we propose a new conditional mechanism to guide video generation and combine it with our model. As mentioned, adjacent frames are strongly correlated in videos. Each frame contains information from frames before or after it. The upper and lower frames can greatly determine what the current frame is like. In other words, to make the model learn adaptively, we can choose some of the frames in the input sequence to guide the whole sequence to learn complete features themselves. Some methods typically used the beginning of continuous frames or the last frames of a video as conditional inputs to guide the generation of current frames [33]. This is well performed in a short period. However, when a longer video needs to be generated, the quality of outputs will quickly decrease, since the correlation among frames gradually weakens as the distance of their positions increases, and these conditions’ information cannot represent all moments, resulting in large generation errors.

To eliminate the impact of temporal distance and ensure the overall quality of generated frames, given $x_{0}$ with *N* frames, we separated $x_{0}$ into *s* bins of equal lengths. Thereafter, we uniformly sampled one frame from each bin. In this way, conditional frames $y=\left[x_{0}^{n_{1}},x_{0}^{n_{2}},\ldots ,x_{0}^{n_{s}}\right]\subseteq x_{0}$ are picked, which contain multiple information and features. The mechanism enables frames in the input sequence to better learn about the previous and the next pattern features and associations among themselves. Also, uniform sampling from each bin allows the model to capture and learn slight pixel-level changes, strengthening the total generation stability.

To learn about the conditional features, we propose a conditional residual sub-block to access conditional guidance. Concatenated conditional frames are passed through a convolution layer and embedded into two learnable tensors $C_{1}$ and $C_{2}$. These two tensors simulate the weight and bias of a linear neural structure to merge with inputs. Specifically, given $x_{t}$ and conditional set y, we have

(6) $$x_{t}=C_{1}\cdot Conv\left(x_{t}\right)+C_{2},$$

where Conv(·) represents convolutional layers. To combine the input with processed conditions, the sub-block uses shortcut connections, which add the input to its convolutional output to deliver information directly and integrate it with refined conditional features. Since the main purpose of this sub-block is to involve incorporated guidance, we used space-only 2D convolutions instead of 3D convolutions to reduce total costs.

**Figure 1:**
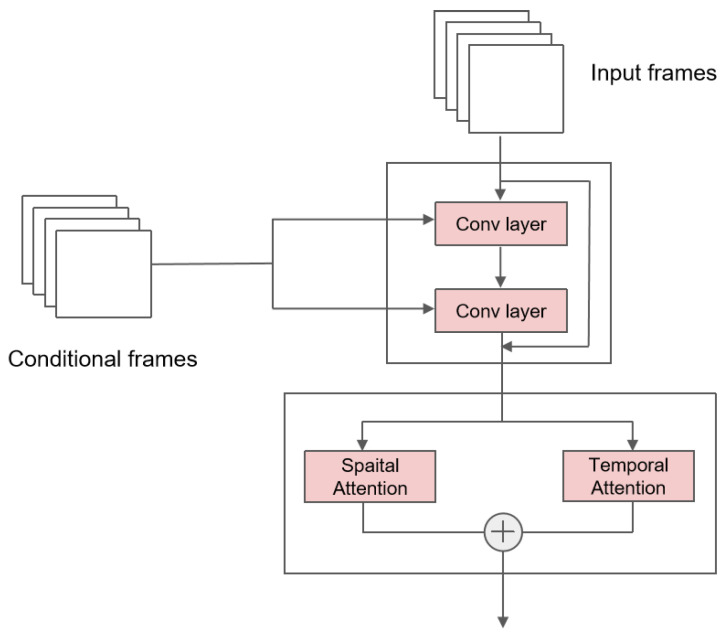
The main block structure of the U-Net. The input is firstly passed through a residual sub-block, where conditional frames are combined with the input via convolutional networks. The output of the residual sub-block is then passed through a spatio-temporal sub-block to learn about inherent features.

Combining the above two sub-blocks, we obtained the main block in the network. The total structure and the details are illustrated in Figure 1. The main block consists of a residual sub-block and attention sub-blocks. The former residual sub-block focuses on combinations of conditions and inputs. The latter multi-head self-attention sub-blocks allow the model to learn about all of the inner-video features well.

### 2.4. Low-Loss Pre-Trained Latent Compression

Traditional methods are inefficient, owing to high computational memory and time costs, especially when the inputs are videos. This constraint makes it difficult for methods to achieve the efficiency requirements of online tasks. Additionally, with the refinement of the structure and the introduction of conditional guidance, our proposed method requires more parameters for training and sampling, which also demand high levels of computational resources, which most ordinary enterprises can ill afford.

To improve efficiency and reduce costs, we propose an efficient compression strategy, which uses a pre-trained autoencoder to transform the full video sequence into a latent space to a high quality. The pre-trained autoencoder consists of two parts. The encoder part E(·) first learns about the non-linear relationship among the inner features of the sequence and encodes them into the latent representation with a down-sampling convolutional network. Thereafter, the decoder part D(·) learns about the reverse mapping from the latent space to the original data and decodes the latent features into final reconstructions ${\tilde{x}}_{0}$ using an up-sampling convolutional network.

In contrast with traditional autoencoders such as VAE, we remove the Kullback–Leibler divergence in the training loss. The Kullback–Leibler divergence aims to summarize the distribution in lower dimensions and avoid high variance [34]. However, its effectiveness is limited in the complex distribution of various scenarios. Furthermore, since the features of different scenarios vary significantly, the Kullback–Leibler divergence will inevitably disrupt the internal features of all the instances while optimizing the training loss, thereby compromising the generation ability. Instead, we opt to scale the encoded frames using min–max normalization Nor(·) for a better convergence of the model, which is based on the maximum and minimum pixel value of frames. During this, frames are projected into lower dimensions, keeping the inherent features of the original structures. Meanwhile, the feature differences between different scenes will not be broken and can be fully learned about by the model. After compression through the encoder part and normalization, we obtain the final latent input:

(7) $$z_{0}=Nor\left(E\left(x_{0}\right)\right),$$

where $z_{0}\in R^{n\times c\times h\times w}$. Since we perform diffusion and denoising in the video latent space, the prior compression makes the process more efficient and less costly for further training and sampling.

### 2.5. Training and Sampling Process

**Figure 2:**
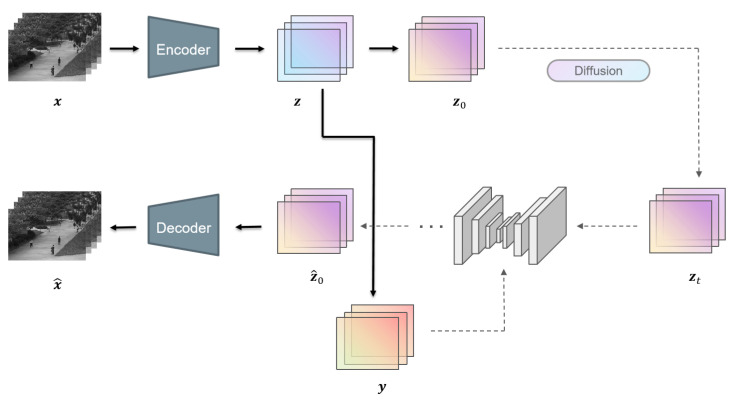
The overall framework of the model. During training, conditional frames y are sampled at first and the encoded inputs z are corrupted via the diffusion process to timestep *t*, sampled randomly from 0 to *T*. During the inference phase, inputs are corrupted into timestep *T* and denoised *T* times. The outputs are then fed into the decoder and reconstructed to the RGB space.

Overall, the whole framework of our proposed method is illustrated in Figure 2. When given a video sequence $x_{0}$, we first put it into a pre-trained autoencoder and obtain the latent input z.

After compression, conditional frames y are selected for further sequence guidance. Thereafter, the latent input is corrupted into Gaussian noises via a *T*-step diffusion process. In this term, parameters are set in advance and all variables can be calculated directly. Hence, the variable after diffusion $z_{T}$ is

(8) $$z_{T}=\sqrt{\bar{\alpha_{t}}}Nor\left(E\left(x_{0}\right)\right)+\sqrt{1-\bar{\alpha_{t}}}\epsilon .$$

Next, we train the model in the reversed process to approximate the distribution. Correspondingly, to cope with different complex distributions for better robustness, we jointly train the conditional model $\epsilon_{\theta }\left(z_{t},t|y\right)$ and unconditional model, which simply sets conditional frames to 0 in the form of $\epsilon_{\theta }\left(z_{t},t|0\right)$ using a binary mask $m_{f}$ with a probability of *p*. The model generates an output with the same dimension as $z_{t}$ to simulate the Gaussian noise as closely as possible. Hence, the loss function is formulated as

(9) $$L\left(\theta \right)=E_{z,\epsilon ,t}∥\epsilon -\epsilon_{\theta }\left(z_{t},t|m_{f}y\right)∥.$$

After training, we obtain simulated noises by measuring the interaction with frame features. By using these noises via the model, we then sample the new video sequences we aim to reconstruct. Unlike generation tasks, VAD more focuses on differences between normal and abnormal events. Specifically, on the premise of the pre-trained autoecoder, we only need to reconstruct the instance in the latent space via the encoder part E(·) and detect the anomaly by simply comparing the discrepancy between original latent inputs $z_{0}$ and reconstructed ones ${\hat{z}}_{0}$. The anomaly error of the *i*th latent frame in a video sequence $z_{0}^{i}$ is calculated by mean squared error as

(10) $$e\left(z_{0}^{i}\right)=\lambda {∥z_{0}^{i}-{\hat{z}}_{0}^{i}∥}_{2}^{2},$$

where λ is a scaling parameter. While sampling, we select the first clip of the video sequence with a sliding window of length *N*. After generation, we shift the window to the next *N* frames and repeat the same operation until the whole sequence is processed.

For better recognition, we use the regularity score based on anomaly error by normalizing the error of each frame as follows:

(11) $$S\left(z_{0}^{i}\right)=\frac{e\left(z_{0}^{i}\right)-e{\left(z_{0}\right)}_{min}}{e{\left(z_{0}\right)}_{max}-e{\left(z_{0}\right)}_{min}},$$

where $e{\left(z_{0}\right)}_{min}$ and $e{\left(z_{0}\right)}_{max}$ represent the minimum and maximum frame error of the whole sequence, respectively. Consequently, errors are normalized into [0,1]. Under this way of calculation, abnormal frames will usually result in higher scores.

## 3. Experimental Analysis

In this section, we evaluate the proposed method on three famous public datasets, including UCSD Pedestrian [35] and CUHK Avenue [36], wherein UCSD Pedestrian has two different subsets, Ped1 and Ped2.

### 3.1. Datasets

The UCSD dataset is a collection of footage from a stationary camera overlooking the sidewalk at 10 frames per second. In this dataset, normal scenes include people walking on the sidewalk. Abnormal scenes include unexpected objects in the regular crowd such as vehicles, bicycles and wheelchairs, or different motion patterns such as riding a skateboard, running and walking on the grass, which are not allowed. This dataset has two different subsets, Ped1 and Ped2. Ped1 has 34 clips for training and another 36 clips for testing. Each clip has 200 frames with a resolution of 238 × 158. Ped2 is split into 16 training clips and 14 testing clips, consisting of 2550 frames and 2010 frames in training sets and testing sets, respectively, with a resolution of 320 × 240.

The CUHK Avenue dataset considers the activities of people at the entry of a station. The dataset has 16 training and 21 testing video clips with a resolution of 640 × 360, and each clip is about one minute long. The training video clips contain more abnormal activities than the UCSD. The anomalies include unusual action patterns like running, randomly standing and throwing objects.

### 3.2. Evaluation Metrics and Setup

In VAD, metrics related to accuracy are widely used to evaluate the effect and performance of the model. Most studies use the receiver operating characteristic (ROC) curve and the area under curve (AUC) to evaluate the results. The ROC curve has the false positive rate (FPR) on the abscissa and the true positive rate (TPR) on the ordinate, which are calculated as

(12) $$FPR=\frac{FP}{FP+TN}\;\;TPR=\frac{TP}{TP+FN},$$

where F and T indicate false and true judgments, N and P indicate negative and positive samples. AUC is calculated as the area under the ROC curve, which represents the ability of accurately predicting all instances. Another metric named Equal Error Rate (EER) is also based on the ROC curve. It is the value of the ROC curve when FPR = 1 − TPR which measures the equal probability of miss-classifying a positive or negative instance. In this study, we adopted AUC and EER based on the testing-video dataset. We labeled each frame in the videos and predicted whether there were abnormalities in frames through the model. These metrics can evaluate the detection effect of abnormal events and are well suited for comparing algorithm performance. Generally, a higher AUC and lower EER represent better model performance.

Before training, the details for setup need to be emphasized. We normalized all frames of the videos to gray scale and then resized their resolutions to 256×256. For each video sequence, the length of the sliding window was set to N=16 and the number of conditional frames was set to s=4. The factor-n encoder composes the original frames from 16×256×256 to $\frac{16}{n}\times \frac{256}{n}\times \frac{256}{n}$, and we set n=4. The learning-rate scheduler and EMA of the model were taken with an initial learning rate of $2.3\times 10^{-4}$ and LinearLR scheduling; weight decay was set to $1\times 10^{-4}$. The network weights were initialized by the “Xavier” method and were optimized by the AdamW optimizer to minimize the training loss.

### 3.3. Anomaly Video-Detection Analysis

Below, model analyses and the effect on benchmarks are given.

For the pre-trained autoencoder, although the method can achieve better results when features are extracted from more complex networks like a 3D autoencoder with a ResNext101 backbone [37], its dimensionality is over 2048, which is large and requires huge training costs. Considering costs and performance comprehensively, we adopted a 3D Autoencoder with a ResNet18 backbone, whose dimensions are only a quarter, without much loss in quality. We also investigated the number of total diffusion steps in the whole process by setting the value to 600, 800, 1000 and 1400. The highest performance is obtained in T=1000 and T=1400 within 1% AUC, while T=1000 requires lower computational costs and a shorter time consumption time for training and sampling than T=1400. Therefore, we adopted the number of total diffusion steps as T=1000 to train and test the model. The model was trained using PyTorch with two A40 GPUs for about four days on each training set.

**Figure 3:**
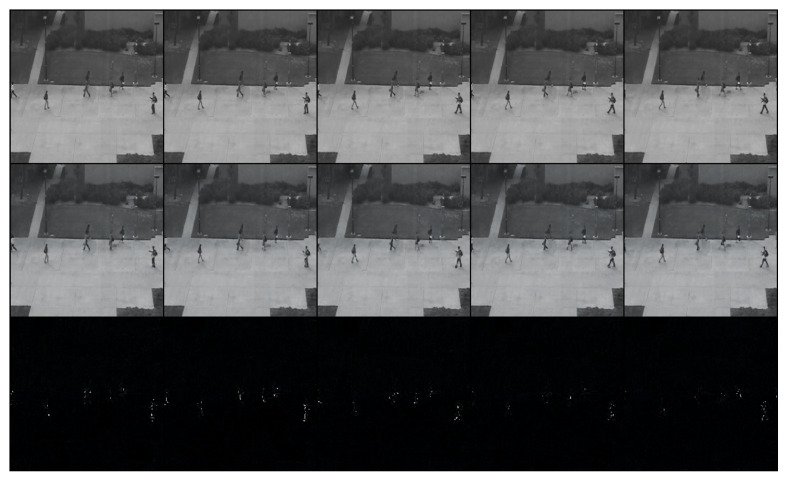
The normal clip of people walking on the sidewalk. The bottom row shows errors between ground truth and normal outputs, which are well fitted as black pixels.

**Figure 4:**
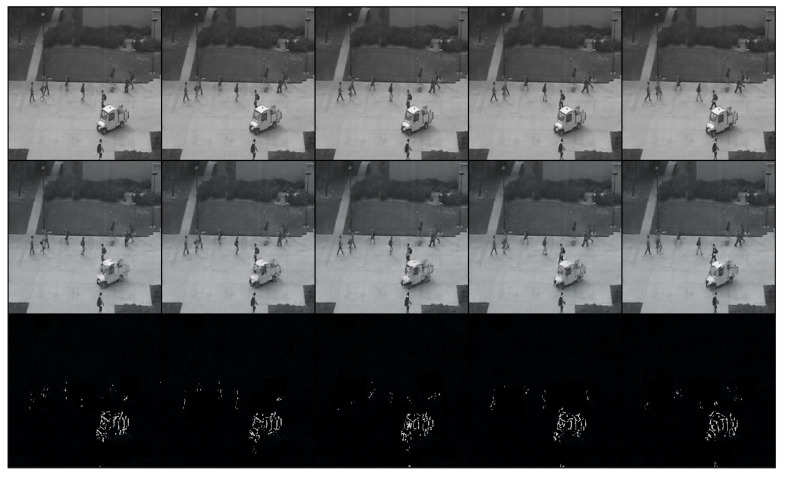
The abnormal clip of a vehicle driving and a man riding a bicycle on the sidewalk. In the bottom row, the vehicle cannot be recognized and caused high errors shown as white pixels.

After training the model, we tested it with different testing-video sequences for intuitive visualization. We show two groups of examples from different scenes in Figure 3 and Figure 4. The rows from the top to the bottom represented ground truth, generated sequences and the errors between them. In Figure 3, people are walking on the road as normal. At this moment, features can be recognized and errors of generation with ground truth are low as shown in the bottom row. Different from Figure 3, a driving white vehicle occurs in Figure 4, which is forbidden in this scene.

Compared with men walking, the proposed method detects noticeably different patterns and objects such as a car driving, which distorts information in the spatio-temporal feature and generates low-quality frames. As shown in the bottom row in Figure 4, the pixel errors of the vehicle are much higher than those of other objects.

**Figure 5:**
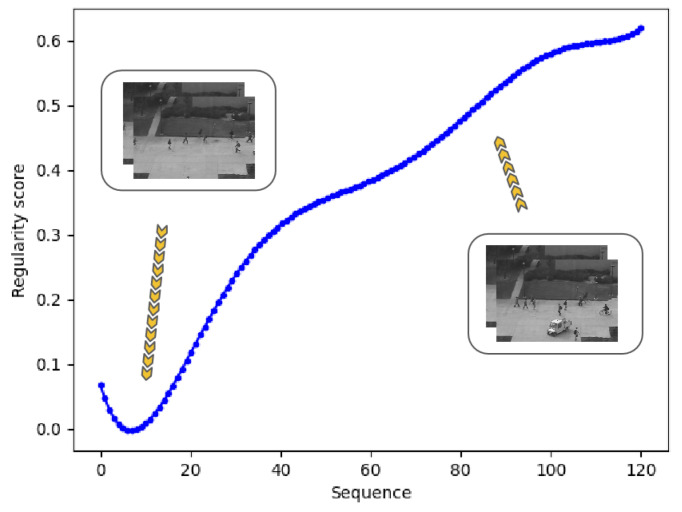
Regularity score curve during a sequence. In normal frames, the score is low, while it becomes high as the car and a man riding a bicycle occur.

The visualization above shows big differences between regular events and abnormal events. Based on this, we used the regularity score to detect abnormal events in video clips. For normal frames, the curve is stable at a low score level. When an abnormal event occurs, the model cannot generate a high-quality frame, resulting in higher errors and regularity scores. The sequences in Figure 5 are from the UCSD Ped2 dataset. People are walking on the sidewalk in the beginning, then a vehicle and a man riding a bicycle appear. Correspondingly, the regularity score curve increases. Based on the changes in the score curve, we can conclude that there are abnormal events in this video clip, which is also consistent with this fact.

### 3.4. Results and Comparison

We compared our method with different SOTA models based on these popular benchmarks. Table 1 provides the performance comparison of the proposed method with the SOTA models, displaying the AUC and EER on each dataset. According to the main concepts of these models, we split them into several groups. Adam [38], MPPCA [39] and Social Force [40] are traditional methods that use handcrafted features. Our method significantly outperforms these traditional methods in terms of AUC and EER. The other methods are deep learning methods, where ConvAE [16], ST-AE [41], STAE-grayscale [42] and ConLSTM-AE [43] are one-stage models which reconstruct data based on an autoencoder, and StackRNN [11] and Attention [44] are more focused on the connection of frame pixels. Additionally, we also considered Ada-net [45], which is a more complex GAN-based model with a higher accuracy.

As shown in Table 1, our proposed method achieves 92.7% in AUC on UCSD Ped1 and 97.4% in AUC on UCSD Ped2, which achieves the best performance compared with others. In the Avenue dataset, our method still attains 88.5% in AUC, outperforming other models by 2.5%. The comparison demonstrates that besides traditional machine learning methods, performances of other unsupervised methods are also inferior to our proposed method. By conditional context guidance, our method fully considers the correlation among frames, which improves self-supervision and makes up for the shortcomings caused by the lack of labels. Our method successfully learns about representative normal features and behavior patterns in multi-modal scenarios and effectively captures unexpected changes beyond normal behaviors. Results on the UCSD dataset demonstrate that our method can successfully distinguish anomalies after training on a certain amount of normal data. For the Avenue dataset, although there are some abnormal data interferences on the training set, our method can better eliminate the negative impact of data noise, which confirms the remarkable effectiveness of latent diffusion-performing VAD.

Although the AUC of our method is high, the EER does not achieve the expectation despite it being competitive. Our method can allow for learning about the realistic distribution of given sequences. Nevertheless, the latent compression strategy unavoidably causes feature loss while improving efficiency. Compared with ST-AE, which trains frames directly, and Ada-net, which uses a high complexity of adversarial networks, our method lacks feature fusions at the pixel level, which leads to a lower stability than the models mentioned above.

## 4. Practical Application

In this section, we verify the performance of our method and the effectiveness of our improvements in a practical application in coal mining.

### 4.1. Coal Mining-Pollution Monitoring

China’s energy structure is continuously evolving and is still dominated by coal and accounts for more than 50% of its primary energy consumption. Over the years, coal-producing cities and surrounding areas have become associated with many environmental issues during the mining, processing and transformation of coal, resulting in significant historical environmental debts. Without legal and effective environmental monitoring and control, the ecosystem will be severely damaged, national production and economy will be affected and even human life and health will be seriously endangered.

Since environmental pollution has always been a global concern, it is necessary to establish relevant pollution-monitoring and -controlling systems in coal-mining sites. The full real-time monitoring of major dust-pollution sources in mining areas is vital to include each type of dust-pollution source in the scope of its supervision. By capturing violation incidents, alarms can be automatically triggered. At the same time, according to management needs, it can automatically activate on-site spraying, fog guns and control methods, transforming passive governance into active governance. This can truly achieve full supervision of dust-pollution sources.

Although many methods have been proposed in the literature to model video data, the practitioners still monitor abnormal dust pollution manually since existing methods require expensive resources for training. Another reason is that the existing methods are inefficient in monitoring anomalous dust pollution quickly and accurately due to the complexity of the models, which have high computational and time costs. Manual monitoring has many limitations. Mistakes are made during monitoring, especially in extreme environments such as at night or during cloudy days, and it is very difficult and inefficient to train personnel.

To provide a basis for environmental law enforcement by improving the grid precision monitoring and intelligent supervision systems for urban dust-pollution prevention and control, and help improve the overall efficiency of dust-pollution prevention and control, it is essential to develop an accurate and efficient framework, which can assist practitioners in monitoring the dust quickly and accurately based on video data. This will help the law-enforcing department make scientific decisions. The main challenge facing this initiative is the failure to capture illegal behaviors. Hence, applying unsupervised VAD methods to environment monitoring is a direction worth exploring.

### 4.2. Anomaly Detection in Video Surveillance

Nowadays, video-surveillance systems can be installed in different industrial or natural scenarios such as factories, construction sites, forests and lakes to collect data. These videos can provide us with a large number of normal samples. By detecting abnormal situations using online models, we can effectively collaborate with relevant departments and initiate corresponding disposal processes to achieve the goal of environmental protection.

**Figure 6:**
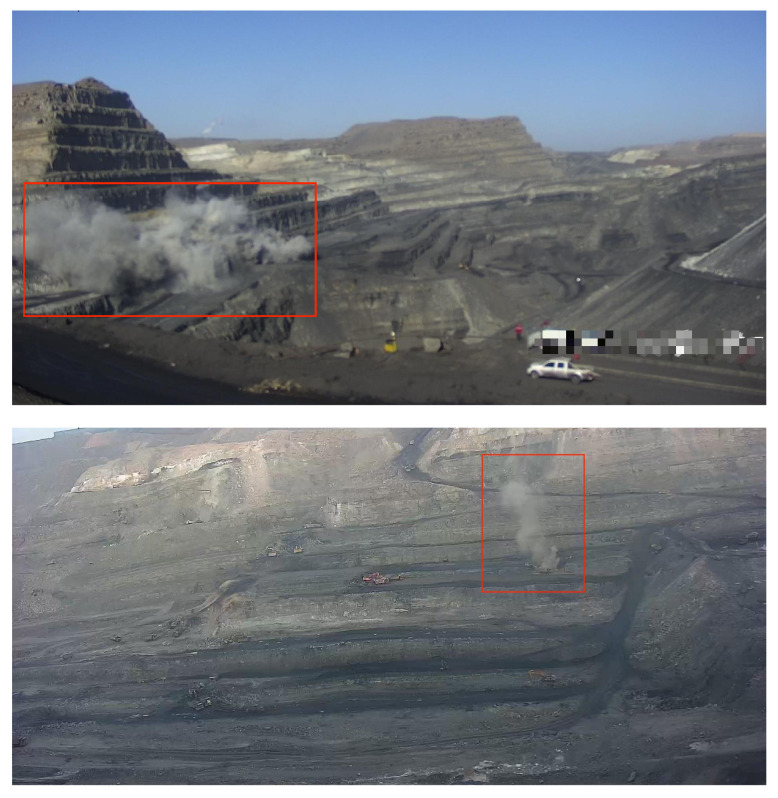
Anomalous frames in the practical dataset. In this situation, polluted smoke and dust marked with red square boxes will only be made by illegal operation or machine breakdown, which are treated as unusual events.

In this practical situation, we applied the proposed method to monitoring. We used a new dataset containing videos from various angles and construction events collected by CCTV cameras covering more than 15 mining areas in a coal-producing city in China that implements daily day and night recording. Abnormal harmful Dust in these mining areas in the dataset are shown in Figure 6. Three frame intervals in the given sequences are available from the dataset: a completely continuous video without frame loss, 5 min between two frames and 10 min between two frames.

We selected more than 20 CCTV systems in mining areas in the same city in northern China to cover real and complex working scenarios. By recording daily video clips from these surveillance systems, we obtained enough data. We divided the videos into short clips of at least 30 and at most 60 frames each. We sifted and split the data into 27 clips for training and 12 clips for testing, respectively. In addition to normal sequences, the training set included some disturbed frames, such as light exposure, an inability to focus the lens and weather changes, whose appearance in real scenes is highly probable. The related model settings stay the same as in the previous section.

### 4.3. Experiments for Validation and Comparison

To validate the performance, we tested our method and compared it with two mainstream models, ConLSTM-AE and Ada-net. Furthermore, we conducted an ablation study with two more models based on our method. One of the models adjusts smaller network hyper-parameters to a half as the proposed lightweight model does to explore the impact of model size. The other model removes the conditional mechanism and replaces the spatio-temporal structure with a spatial-only structure while the rest of the parts remain the same, which corresponds to our improvements in the proposed architecture. These two models were trained and compared with the proposed full model we adopt currently. Results are shown as follows.

**Figure 7:**
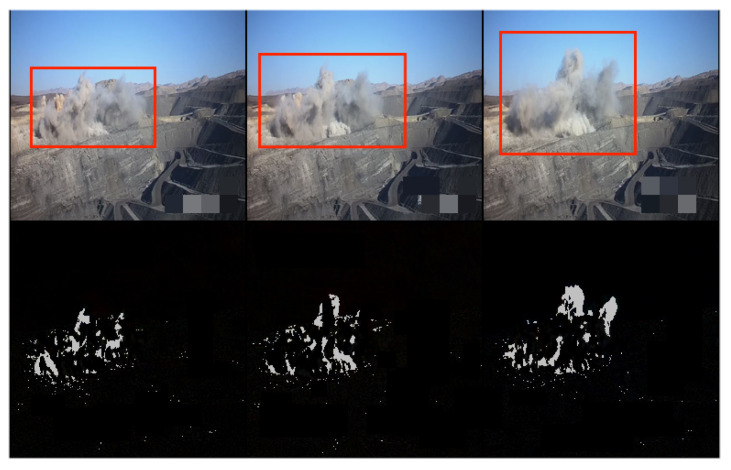
The effect visualization of the proposed method in the abnormal coal-mining surveillance. The upper row is the original video sequence with abnormal polluted dust marked with red boxes, and the bottom row is errors of frames between the original and reconstructed ones.

In Figure 7, the red boxes in the upper row indicate the abnormal polluted dust in the surveillance systems, and the white pixels in the bottom row represent the abnormal areas identified by the proposed method, which are bounded by the red boxes. It can be seen that our proposed method successfully identifies most of the abnormal dust in the video sequence. From Table 2, our method achieved 73.6% in AUC, which outperforms the current models, and 28.2% in EER, which is competitive with the compared models.

The results confirmed the excellent recognition performance of the proposed method once again. It should be noted that since our dataset contains a variety of complex scenarios and parts of abnormal sequences, the difficulty of the model training and sampling significantly increases, leading to a foreseeable deterioration in AUC and EER compared with the public datasets in the last section.

**Figure 8:**
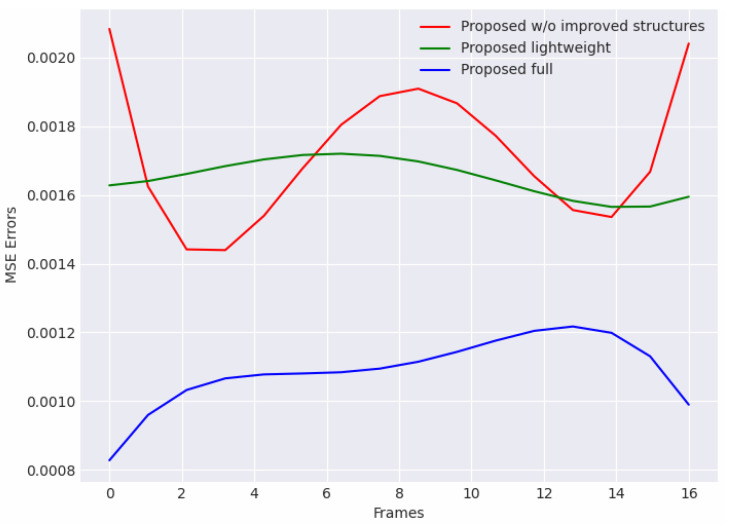
Reconstruction error curves of models in the ablation study which were curved in a 16-frame clip in normal video surveillance. The proposed full model obtains the lowest errors in all three compared models.

Compared with other proposed models in the ablation study, it can be found that both the other two models perform worse. As shown in Figure 8, we curve the MSE reconstruction errors of three models in a certain clip, which is randomly picked in normal video sequences. The propose full model better learns distributions of normal scenarios, and the reconstruction errors in the normal clip is significantly lower than other two models.

On the other hand, although the errors of the proposed model without improved structures are not much different from those of the lightweight model, their error variance is larger. This indicated that our improvements can enhance the consistency and stability of the model for continuous frame reconstructions in input sequences. Each frame in the sequence is well learnt and guided. In Table 2, the performance of the model without improved structures is also significantly worse. The difference is up to 5.3% AUC, which verifies the effectiveness of our improvements. Meanwhile, the proposed lightweight model shows a lower AUC and a higher EER than the proposed full model. The result indicates that the high-accuracy performance of the method requires the sufficient support of learnable model hyper-parameters. However, it should be noticed that although the lightweight model performs worse in its accuracy, its sampling speed is about one-third faster than that of the full model, which is highly important in real-time monitoring. This shows a possible improvement in which the model can trade off some acceptable performance in accuracy for higher efficiency, and provides a direction for better hyper-parameter exploration in practical monitoring.

Unlike the public benchmarks mentioned above, the recognition of multiple scenes and the interference of practical natural factors increase the difficulty of model training. However, our method can still identify the occurrence of abnormal phenomena in most scenes. Our model successfully detects rising dust with high errors while other normal parts like mountains and the sky remain as low errors. This study verifies the feasibility of the practical recognition of various situations to some extent and provides a direction for further applications in VAD.

## 5. Conclusions

In this study, we introduce a new unsupervised method based on latent diffusion in VAD. We proposed a new network architecture that incorporates a conditional guidance mechanism and spatio-temporal structure to fully leverage the inherent features of instances and generate high-quality reconstructions. Additionally, we employed an improved latent compression strategy to reduce costs without sacrificing the original video’s feature integrity. To validate the performance of our method, we conducted experiments and comparisons on three popular public benchmarks. Our experimental results demonstrate that our method effectively enhances the accuracy and stability of video-anomaly detection outperforming current SOTA models.

Furthermore, we applied our method to a practical application in coal-mining scenarios which may produce dust pollution, and discussed its performance across several studies. The results in practical application studies verify the effectiveness of our method in handling different complex scenarios in environmental monitoring, providing accurate alarms and assisting in enhancing law-enforcement efficiency. Our method effectively learns about the distribution of video data in complex scenarios and performs efficient generation and anomaly detection with lower computation costs.

Despite the excellent performance of the model, it is noticed that there are still some limitations that need to be addressed. First, the current regularity score is not suitable for scenarios where there are no abnormal events within a certain period, as the model may misidentify normal sequences as being abnormal. Second, the issue of high time costs has not been fully resolved. Although latent compression improves efficiency, the diffusion process is still time-consuming, with hundreds of steps or more involved. Future work includes improving generalizability in multiple complex scenarios, exploring more efficient patterns in the generation and detection process, and refining the framework to reduce misjudgments in longer windows of normal scenes, which are crucial for practical implementation in real-life problems. Exploring our applications in other potential domains, such as natural disasters and traffic accident monitoring, is also an interesting theme for further research.

## Figures and Tables

**Table 1:** Comparison in AUC and EER of different methods in benchmarks, where “–” indicates that the value is not published or provided in corresponding articles. The values in bold font indicate results of our method, which are better than others.

Dataset	Ped1		Ped2		Avenue	
Method	AUC (%)	EER (%)	AUC (%)	EER (%)	AUC (%)	EER (%)
Adam [38]	77.1	38.0	–	42.0	–	–
MPPCA [39]	66.8	40.0	69.3	36.0	–	–
Social Force [40]	67.5	31.0	55.6	42.0	–	–
ConvAE [16]	81.0	27.9	90.0	21.7	70.2	25.1
ST-AE [41]	89.9	12.5	87.4	12.0	80.3	20.7
ConvLSTM-AE [43]	75.5	–	88.1	–	77.0	–
STAE-grayscale [42]	92.3	15.3	91.2	16.7	77.1	33.8
StackRNN [11]	–	–	92.2	–	81.7	–
Attention [44]	83.9	–	96.0	–	86.0	–
Ada-net [45]	90.4	15.8	90.3	15.5	83.5	23.5
Ours	**92.6**	18.3	**97.4**	20.2	**88.5**	24.7

**Table 2:** Performance comparisons in the testing dataset of coal-mining scenes. * indicates our implementation since the corresponding code is not publicly available. The values in bold font indicate the best results in comparison.

Method	AUC (%)	EER (%)
ConvLSTM-AE *	59.7	34.3
Ada-net *	70.3	**25.9**
Proposed w/o improved structures	69.3	30.2
Proposed Lightweight	70.8	28.7
Proposed full	**73.6**	28.2

## Data Availability

Restrictions apply to the availability of these data.
